# Current Evidence for Sleep States in *Drosophila*: Findings and Implications

**DOI:** 10.1007/s40675-025-00352-w

**Published:** 2026-03-27

**Authors:** Maria E. Colt, Susan T. Harbison

**Affiliations:** Laboratory of Systems Genetics, National Heart Lung and Blood Institute, National Institutes of Health, Bethesda, MD USA

**Keywords:** Sleep states, Drosophila, Microbehaviors, Neural correlates, Genetic networks, Sleep stages

## Abstract

**Purpose of Review:**

Sleep is an essential biological behavior, with its absence leading to severe consequences, including death. In mammals, sleep consists of distinct states—such as REM and non-REM—that are often thought to serve different physiological functions. Traditionally, *Drosophila melanogaster* were believed to experience sleep as a single, unitary state. However, recent research suggests that sleep in flies is more complex than previously understood and can be divided into distinct states. This raises the possibility that the fly model can be used to investigate the functional role(s) of each sleep state.

**Recent Findings:**

In this review, we explore the behavioral, neurophysiological, metabolic, and transcriptional evidence supporting the existence of these sleep states in *Drosophila*. We assess whether consistent criteria can be established for these sleep states and propose a new direction for sleep research by identifying genetic correlates associated with these states.

**Summary:**

This approach has the potential to deepen our understanding of sleep architecture and its genetic underpinnings, offering insights that may extend beyond the *Drosophila* model to other species, including humans.

## Introduction

Sleep is a fundamental biological process observed across nearly all animals studied [1], and its importance is underscored by the severe consequences of both acute and chronic sleep deprivation which can lead to performance and cognitive impairments and even death [2, 3]. Over the past two decades, the fruit fly, *Drosophila melanogaster*, has proven to be a valuable model for sleep research due to its genetic tractability. The genetic tools available in *Drosophila* have facilitated studies aimed at understanding the causes and consequences of sleep through both forward and reverse genetic approaches [4]. A wealth of knowledge has been gained regarding the genetic networks underlying and regulating sleep, along with the identification of the neural circuitry where these genes act [4]. However, a complete understanding of the genetic mechanisms underlying sleep remains elusive due to the complex genetic architecture of sleep, its regulation by genes having pleiotropy with normal physiology and disease, and its susceptibility to change with environmental perturbations [5, 6]. Notably, sleep in *Drosophila* has traditionally been regarded as a unitary state, characterized by periods of inactivity lasting at least five minutes [7].

Recent studies suggest that, much like in humans [8], fly sleep may be divided into distinct states each having specific behavioral and electrophysiological characteristics. Initial investigations into *Drosophila* sleep states used arousal threshold testing to measure responsiveness, under the assumption that flies in deeper sleep states are more difficult to awaken [7, 9–11]. Additional research revealed associated changes in microbehaviors (See description below in “Distinct microbehaviors reveal sleep states”) [12, 13], local field potentials (LFPs) [11–15], neural calcium [16] and voltage activity [17], gene expression [18], and metabolic rate [19] during sleep. Computational modeling and machine learning approaches also showed complexity in sleep architecture [12, 20, 21]. If these characteristics map to varying sleep depths, the question arises as to whether the efficacy of the *Drosophila* genetic model can be used to identify and characterize the genetic and physiological basis of different sleep states, which will lead to a better understanding of their functional consequences.

In this review, we outline the current evidence for the presence of multiple sleep states in *Drosophila* and discuss the implications of these studies. We also highlight the challenges of defining sleep states in flies and offer a potential path forward for the field, providing insight into how we can leverage the *Drosophila* model to understand the genetic underpinnings of sleep architecture.

## Emerging Evidence that Flies Have Multiple Sleep States

### Changes in Arousal Threshold Occur During Sleep

A long-accepted criterion for sleep is reduced behavioral responsiveness [22], which has been observed in *Drosophila* by applying tapping, heat, or light stimuli during rest. Early studies demonstrated that resting flies exhibited reduced responsiveness to tapping, making it more difficult to rouse a sleeping fly compared to an awake one [7, 10]. Shaw and colleagues [7] detected a five-minute immobility threshold for reduced arousal, while Hendricks and colleagues [10] noted that both the number and intensity of tapping stimuli required to wake flies increased during the night. The notion of a five-minute immobility threshold was supported by additional studies using a variety of stimuli in several wildtype strains (Table 1) [14, 15, 23, 24]. This work led to the widespread application of five minutes of immobility as the threshold demarcating sleep from wake in flies—with sleep interpreted as a unitary state.

**Table 1.**
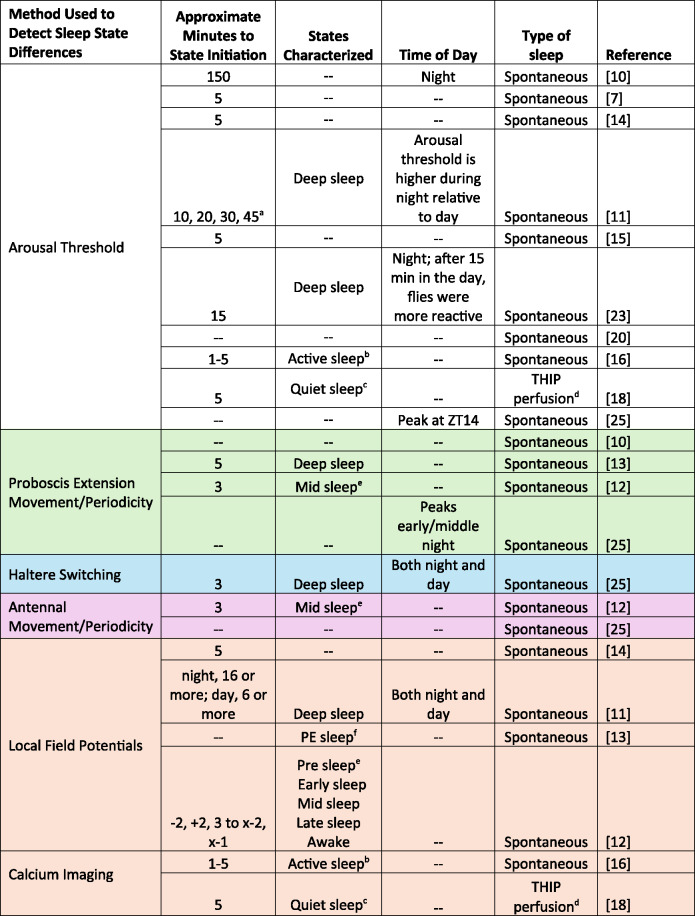

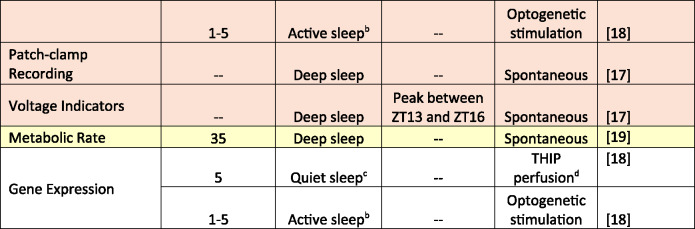
Summary of methods used to detect sleep states and findings. Colors correspond to Fig. 1 external bodily structures

^a^Flies achieved a deeper sleep state around 10 min; sleep became lighter at 20 min; a second deeper state appeared at 30 min; then sleep became lighter at 45 min. ^b^Active sleep refers to flies that are behaviorally quiescent with increased arousal thresholds and yet have wake-like brain activity. ^c^Quiet sleep refers to flies that are behaviorally quiescent with increased arousal thresholds and decreased neural activity. ^d^THIP, 4,5,6,7-tetrahydroisoxazolopyridin-3-ol. The drug was perfused directly to the brain. ^e^Pre sleep occurs 2 min before the start of the sleep bout; Early sleep occurs within the first two minutes of a sleep bout; Mid sleep occurs from the end of early sleep until 2 minutes before the end of the sleep bout; Late sleep occurs in the last 2 minutes of the sleep bout.^f^PE, proboscis extension. PE sleep is characterized by decreased activity in the brain.

Studies taking a more systematic approach to measuring arousal threshold during sleep revealed an unexpected complexity in the sleep architecture of flies: flies had distinct sleep states rather than a unitary sleep phase [11]. Applying a mechanical stimulus to the flies, Huber and colleagues observed a progressive decrease in responsiveness between 1 and 5 min of immobility, followed by a plateau in responsiveness after 5 min of immobility that persisted up to 12 min [24]. Likewise, subsequent work showed that responsiveness began to decrease after one min of immobility and reached a low point between 5 and 10 min [11, 16]. Intriguingly, flies became more responsive after about 20 min of immobility, and again were less responsive around 30 min of immobility, followed by increased responsiveness between 45 and 60 min after sleep onset [11]. A computationally-derived probability of waking that varied throughout the day likewise corresponded to shifts in mechanical or light-induced responses [20]. Additionally, an infrared laser used to heat the flies revealed a correlation between stimulus level and the amount of time the fly was inactive [25], with the highest stimulus level needed at zeitgeber time (ZT) 14 (i.e., early night) and decreasing until ZT23 (i.e., late night). These studies highlight the previously underappreciated complexity of sleep architecture in *Drosophila*, including the possibility of multiple sleep states.

### Distinct Microbehaviors Reveal Sleep States

**Fig. 1:**
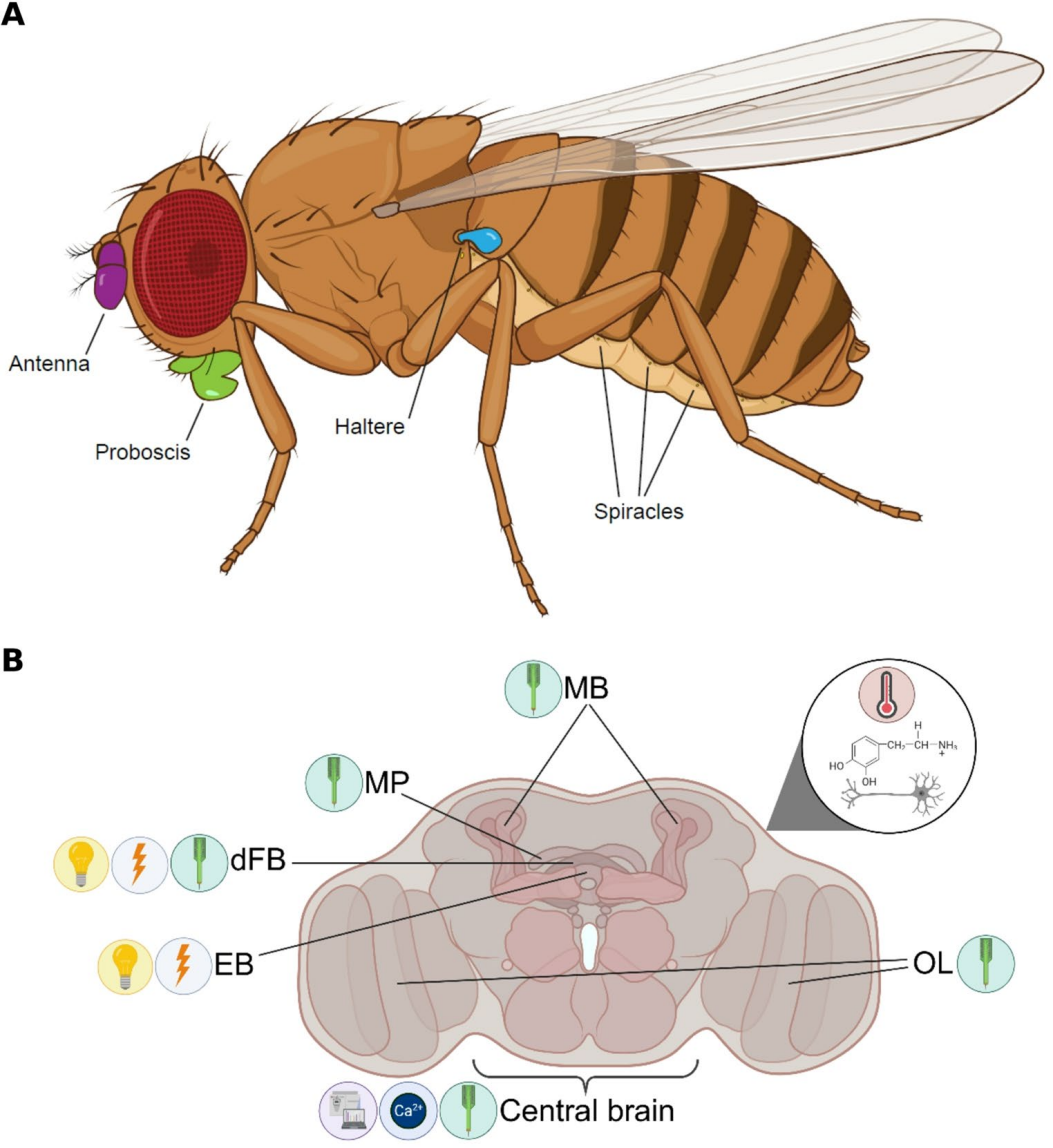
The external bodily structures and brain regions used to define sleep states in *Drosophila*. (**a**) The highlighted external structures (i.e., antenna, proboscis, haltere, and spiracles) are crucial for observing microbehaviors and metabolic alterations indicative of distinct sleep states. Created in BioRender. Harbison, S. (2025) https://BioRender.com/k92rzfs. (**b**) Brain regions include the EB (ellipsoid body) [17, 25, 26], dFB (dorsal fan-shaped body) [15, 25, 26], MP (medial protocerebrum) [14], MB (mushroom bodies) [11, 13, 14], OL (optic lobes) [11–15], dopaminergic neurons (illustrated in magnified circle) [20], and central brain [9, 12, 15, 16], which are essential for observing alterations in brain activity that help define sleep states. Icons represent the methodologies employed: the green probe indicates local field potentials, the yellow light indicates optogenetic activation, the red thermometer indicates thermogenetic activation, the orange bolt indicates voltage imaging, the blue Ca^2+^ indicates calcium imaging, and the purple mass spectrometer indicates a MALDI-TOF mass spectrometer. Created in BioRender. Colt, M. (2025) https://BioRender.com/w8eplb4

Arousal threshold measures were not the only evidence of multiple sleep states. Early on, Hendricks et al. [10] observed postural drooping, respiratory pumping, and proboscis, abdominal, and extremity movements during sleep. Enabled by advanced video recording technology, later studies found that these patterns of brief movements, often referred to as microbehaviors, mapped to different sleep states. Several types of movements have been quantified: (1) proboscis extension, which is an extension and retraction of the fly’s mouthpart in the absence of food; (2) movement of the antennae; and (3) switching the position of the haltere, a balancing organ located behind the wing (Fig. 1A). Sleep could be divided into different states based upon periods of reduced and increased numbers of proboscis extensions [13]. Specifically, sleep featuring greater numbers of proboscis extensions was associated with a reduced response to a vibrational stimulus, indicating a deeper state of sleep. Similarly, periodic movements of both the proboscis and the antenna increase during sleep, with the highest number of events coinciding with deeper sleep [12]. Lastly, the fly’s haltere tends to droop as it enters deep sleep [25]. These microbehaviors serve as valuable indicators to identify different sleep states in *Drosophila*.

### Sleep States have Distinct Correlates of Neural and Physiological Activity

Sleep in *Drosophila* is not only marked by behavioral changes, but also by distinct neural and physiological alterations. Pioneering studies on the *Drosophila* brain identified specific LFPs [11–15], as well as neural calcium [16] and voltage imaging [17], associated with varying sleep depths. Additionally, changes in the expression of specific genes [18] and in metabolic rate [19] have been linked to particular sleep states. An early study [14] revealed that resting is associated with decreased spike-like LFP activity in differential recordings between the medial protocerebrum and the optic lobe of the brain (Fig. 1B), suggesting that shifts in neural activity correspond with waking and sleep states. This finding was bolstered by subsequent research showing that LFP activity decreased during sleep [11–13]. Further insights came from the ArcLight voltage indicator [17], revealing slow-wave-like electrical activity in R5 neurons within the ellipsoid body of the central brain that initially increased early in the dark phase (ZT 13–16) but diminished as sleep progressed. Furthermore, GCaMP6f, a calcium indicator, increased activity during the first five minutes of quiescence, followed by reduced activity thereafter, along with significantly decreased connectivity among active neurons during sleep compared to wakefulness [16]. This observation suggests a fundamental difference between the first five minutes of quiescence and the remainder of sleep. Additionally, a correlation between metabolic rate and sleep exists, with deeper sleep corresponding to reduced metabolic rate [19]. Collectively, these neural and physiological traits underscore the complexity of sleep as a behavior and offer new tools for characterizing sleep states in *Drosophila*.

## Responsiveness to Stimuli Varies Across the Circadian Day

Notably, recent research has highlighted differences in behavioral and physiological responsiveness between daytime and nighttime sleep. Flies, like humans, are diurnal and sleep predominantly through the night. The percentage of flies responding to vibration stimuli decreased during the night compared to the daytime, and simultaneously, lower LFP frequencies occurred in the 11–40 Hz range during the night [11]. Also, metabolic rate was higher during the day and lower at night [19]. Furthermore, as the period of immobility lengthened, daytime sleep became lighter, whereas nighttime sleep grew deeper [23]. This suggests fundamental differences between sleep that occurs during the day and sleep that occurs during the night, a feature of sleep supported by the relatively low genetic correlations between day and night sleep parameters [4].

Beyond differences between night and day sleep, other investigations have noted increasing and/or decreasing arousal thresholds throughout the night. Hendricks et al. [10] found that the total amount of stimulation required to wake flies increased significantly from the first 2.5 h to the last 6.5 h of the night. Proboscis extensions, which correlate with deeper sleep, were most frequent at the beginning of the night (ZT12), decreasing as the night progressed [13]. Arousal threshold, as assessed with infrared laser, was highest around ZT14 [25]. Raccuglia et al. [17] observed rhythmic oscillations from a genetically encoded voltage indicator that targeted R5 neurons between ZT 8–12 and ZT 0–3, peaking between ZT13 and ZT16. Intriguingly, these rhythmic oscillations occurred in the delta (i.e., less than 4 Hz) frequency range, which the authors defined as slow wave activity. Building on this, recent work demonstrated that synchronized slow-wave activity at night that occurs between sleep-promoting and locomotor neurons dampens sensory responsiveness to visual stimuli, revealing a mechanistic basis for sensory disconnection during sleep [26]. At the same time, strong sensory stimuli were still capable of overriding this dampening effect [26]. These studies suggest that sleep may deepen in the first portion of the night and possibly transition to lighter states in the latter portion of the night.

## What Duration of Inactivity Corresponds to Deep Sleep?

Several studies have identified specific durations at which quiescent flies transition into distinct sleep states. Initially, it was shown that flies quiescent for five or more minutes were less responsive to vibration stimuli [7], a finding confirmed by subsequent research [15, 24]. This five-minute threshold for entering sleep has been established by multiple studies, correlating proboscis extensions and increased arousal thresholds with inactivity of five minutes or more [13], and observing reduced LFP following stimuli in flies quiescent for this duration [14]. Furthermore, a wake-like sleep state was defined between one and five minutes of inactivity and a deeper state at five or more minutes [16]. These distinct behavioral and physiological changes may indicate transitions into deeper sleep states.

Other studies have reported longer thresholds for entering sleep states, highlighting inactivity durations as potential markers for different sleep states. In an early study, immobility periods lasting one minute or less were excluded from the definition of sleep, noting that most sleep occurs in bouts longer than 30 min [10]. Flies asleep for 16 to 20 min exhibited significantly lower 11–40 Hz sigma power, and arousal threshold experiments suggested that deeper sleep is achieved after approximately 10 min of immobility [11]. After this deepest sleep intensity, responsiveness levels fluctuate, with lighter sleep occurring around 20 min and a second deep stage emerging around 30 min [11]. Sleep then lightens again, as flies quiescent for 45 to 60 min become as responsive as those quiescent for less than five minutes [11]. Modeling suggests that the deepest sleep occurs around 12–15 min after becoming quiescent [11]. The proportion of flies responding to stimuli decreased gradually during the first 15 min of immobility [23], and a significant reduction in metabolic rate was observed after 35 min of sleep [19]. Moreover, it has been suggested that deeper sleep occurs after 25 min or more of quiescence [9], and the timing and characteristics of four distinct states have been clearly defined (i.e., presleep, early sleep, mid sleep, and late sleep) [12]. Taken together, these studies suggest that flies transition between multiple sleep states throughout the night and point to a five-minute duration marking the onset of sleep, while longer periods such as 15 min and 30 min appear to signal transitions into deeper sleep states.

## Two or More Sleep States Likely Occur in Flies

Despite accumulating evidence of temporal sleep-associated phenomena, quantifying the number of sleep states in flies remains challenging, given that sleep is a complex, continuous behavior. Increased oscillatory LFP activity during sleep induction, followed by a state of desynchronized or decreased brain activity [15], along with calcium imaging studies, suggested the presence of at least two distinct sleep states beyond waking [16]. The identification of sleep periods having numerous proboscis extensions and sleep periods without them also implied at least two states of sleep, with high numbers of proboscis extensions signaling the deepest state [13]. Using a Hidden Markov Model, sleep and activity data have been categorized into four putative states, including deep sleep, light sleep, early wake, and full awake [20]. Combining electrophysiological and microbehavioral analyses suggested four sleep states in addition to waking including presleep, early sleep, mid sleep, and late sleep [12]. While other studies did not speculate on the total number of sleep states, they indicated that flies seem to be in a deeper state of sleep after 10 min compared to one min [11], and also implied the existence of two sleep states in addition to wake [19]. Overall, the evidence indicates the potential presence of at least two distinct sleep states, providing a framework for future research to further clarify the definition of these states.

## Each Sleep State may Serve a Different Purpose

Much like human sleep, there appears to be support for multiple functions of sleep in *Drosophila*. Various hypotheses have been proposed suggesting that sleep may play crucial roles in memory consolidation and reconsolidation [8, 27–29], learning [8, 30], synaptic and cellular homeostasis [8, 30], energy allocation [27, 30], immune system regulation [31], and waste clearance [32]. Although many studies on *Drosophila* sleep states have not directly addressed these questions, some have proposed potential functions. Authors noted that the 7–10 Hz LFP oscillations observed during *Drosophila* sleep resemble mammalian sleep spindles typically seen in non-rapid eye movement sleep [15]. Likewise, slow-wave oscillations in flies might serve a similar purpose to those in mammals, signaling sleep pressure [17] and promoting sensory disconnection during sleep [26]. The deeper sleep state, marked by reduced metabolic rates, may represent an evolutionarily adaptive mechanism for conserving energy [19]. Additionally, the extension and retraction of the proboscis during sleep may facilitate waste clearance from the brain [13]. This work supports current hypotheses on the function of sleep.

The function of sleep was further investigated by inducing different sleep states. In *Drosophila*, “active” sleep refers to a wake-like sleep state characterized by heightened neural activity and decreased responsiveness, and it is conceptually analogous—but not identical—to REM sleep in mammals [15]. Short sleep bouts of 1 to 5 min resemble these wake-like or active states, where flies are unresponsive to visual and mechanical stimuli, suggesting these bouts reflect a form of active sleep [16]. This active form of sleep could be replicated experimentally by stimulating dorsal fan-shaped body neurons [16, 18]. RNA-seq of brains where the active form of sleep was induced optogenetically revealed the upregulation of genes involved in axonal guidance and synaptic vesicle recycling [18]. Anthoney et al. [18] contrasted these findings with gene expression profiles of brains where a deeper form of sleep had been induced via the drug THIP. They found a downregulation of genes involved in metabolism [18]. In addition to revealing the potential purpose of lighter and deeper sleep states, these changes in gene expression could be used as markers to identify these states.

## Commonalities Across Methodologies to Define Sleep States

These studies have utilized a range of methodologies to identify distinct sleep states in *Drosophila*, so it is perhaps not surprising that the field has not yet reached a consensus on the criteria defining these states. Despite this, several commonalities emerge that could be used to characterize states. Notably, multiple studies identified the initial minutes of sleep, typically around one to five minutes, as a critical marker for changes in neural activity and responsiveness [12, 13, 16, 18]. This observation suggests that these brief periods of inactivity are crucial for recognizing transitions into deeper sleep states. Longer sleep bouts are often linked with deeper sleep states, characterized by higher arousal thresholds and decreased responsiveness [23, 33]. Moreover, a recurring theme is the increase in arousal threshold as sleep progresses, signifying deeper sleep states [10, 23, 25]. Proboscis extensions appear more frequently during these deeper sleep states [12, 13]. Studies employing electrophysiology and neural imaging have demonstrated that specific neural activities, such as LFP oscillations, are associated with varying sleep states and depths [15, 18]. The consistent observation of oscillations within the 5–10 Hz frequency range suggests that this range may play a significant role in characterizing *Drosophila* sleep states [12, 15].

Despite this, discrepancies in the results indicate that other factors may influence sleep states. One such factor is genotype, which strongly influences sleep parameters [4]. Sleep traits typically have moderate heritability and are influenced by large and often sex-specific genetic networks [4]. Another factor is the salience of environmental stimuli and context dependency of sleep. It has been shown that sleeping *Drosophila* react to salient olfactory stimuli [34] and vibrations can induce sleep rather than stimulate movement [35]. Another factor is the type of sleep that flies are experiencing – whether spontaneous or induced (Table 1). Induced sleep, achieved through methods like optogenetics or pharmacological intervention with Gaboxodol, often displays different characteristics compared to endogenous sleep, underscoring the need to factor these conditions into experimental designs. To advance the field, the *Drosophila* sleep community must agree on standardized definitions and criteria for sleep stages, considering both genetic influences and environmental factors.

## Challenges

Considerable evidence supports the view that *Drosophila melanogaster* have more than one sleep state. Orthogonal methods—the behavioral response to stimuli, the presence of microbehaviors, the observations of changes in local field potentials and neuronal imaging, the variation in metabolic rate, and the differences in gene expression between different types of sleep—all point to the existence of lighter and deeper sleep states in flies. These observations present a quandary for a field that spent the last two decades identifying genes, gene networks, and neuronal substrates that impact sleep traits derived under the assumption that sleep in flies is a unitary state. Can the field reconcile its previous findings with these new observations? We believe it is possible to incorporate the earlier research into the framework of multiple sleep states and to continue the innovative work cited here to reveal the genetic processes underlying each state. Doing so will enable us to gain an understanding of the meaning and function of each distinct sleep state using this powerful genetic model organism.

A short-term goal for *Drosophila* researchers is to reach a consensus on the number of sleep states and their distinguishing characteristics. As we have shown, evidence thus far distinguishes two to four distinct sleep states in flies [11–13, 15, 16, 19, 20]. To some degree, the number of states identified depends on the criteria used to distinguish among states, and currently disparate approaches are used. Thus, a related goal is to develop robust criteria to identify these states. Jagannathan et al. [12] have made the first attempt thus far to define state-specific criteria. As additional attempts to define and replicate these measures emerge, the consensus will be revealed.

A second goal is to map sleep state parameters to the genome. EEG patterns are known to be highly heritable in humans and mammals [36–39], and the time spent in lighter and deeper stages of sleep is also heritable in humans and other mammals [39–44]. Thus, we anticipate that parameters describing sleep states in flies will be heritable as well. Any heritable parameter can be mapped to the fly genome. Here the potential choice of methods are limited only to the researcher’s imagination and include forward and reverse mutant screens, quantitative trait locus mapping, genome-wide association studies (GWAS), selective breeding, and transcriptomic, proteomic, and metabolomic approaches [4]. This is a major strength of the *Drosophila* model: the ability to apply extant fly mutant and wildtype resources to identify genes in any phenotype of interest. High-throughput measurements can be made quickly and inexpensively compared to other model systems.

At the current level of technology, a high-throughput genetic survey would be difficult using LFPs, calcium signaling, and voltage indicators due to the challenging technical nature of these experiments. While these techniques are becoming more accessible [45], advances in technology are needed to make these measures more high-throughput. An alternative approach might be to use other indicators of sleep states as a proxy for changes in neuronal signaling. For example, one could quantify numbers and patterns of microbehaviors using high-resolution videography to derive sleep state parameters [25]. An additional approach may be to use computational modeling techniques on conventional activity count or video data to quantify parameters around sleep states [20, 21]. It is possible to apply the Hidden Markov Model, for example, to quantify the length of time spent in a sleep state, the number of bouts for a given sleep state, and the average bout length of a given sleep state (Ghosh and Harbison, unpublished data). One could also use a single heuristic, such as a 25- or 30-minute definition of sleep, to target genetic screens for deeper sleep states, as has been suggested by Abhilash and Shafer [[9]; Abhilash and Shafer, *BioRxiv*, doi: 10.1101/2025.06.23.661143]. These proxy measures could be used to estimate the heritability of sleep state parameters and map them to the genome. Importantly, heuristics and HMM modeling could also be used to re-analyze historical *Drosophila* Activity Monitoring System data to determine whether genes identified previously perturb a particular sleep state. A crucial step will be to link the function of candidate genes derived from studies of proxy phenotypes to changes in neuronal signaling and behavior that define changes in sleep state; an example of this was the demonstration that NMDA receptors play a crucial role in the synchronization of R5 neurons [17].

A final goal is to determine whether genes affecting sleep state changes in flies have orthologs in humans with similar functions using cross-species analyses. This is a challenge that has been met using summary sleep parameters such as sleep duration and bout length [46–51]. Researchers have identified genes in flies and zebrafish and demonstrated their conserved functional role in mammals and humans [46–50, 52–54]. The opposite approach has also been taken: beginning with the human GWAS data or mammalian mutational screens, functional tests of candidate genes in flies and zebrafish have been used to explore mechanisms impacting sleep [55–58]. These approaches are fundamental to gaining an understanding of human sleep and sleep disorders.

In tackling these challenges, the *Drosophila* model not only enriches our knowledge of sleep architecture, but also provides a valuable link to genetic mechanisms relevant to human sleep stages. As we refine methodologies within the research community, we can advance our understanding of the genetic factors influencing sleep states offering insights with implications across species.

## Conclusions

Recent studies on *Drosophila* sleep have uncovered distinct sleep states, challenging the long-held belief that sleep in flies is a unitary state—an unexpected discovery that reshapes our understanding of sleep architecture in this model organism. By mapping the timing and fluctuations of behavioral, neurophysiological, and metabolic traits, researchers have identified microbehaviors and changes in arousal thresholds, local field potentials, metabolic rates, and gene expression, all pointing to the existence of lighter and deeper sleep states. These findings highlight the complexity of sleep and suggest that flies transition through multiple states, each with unique characteristics and potential functions.

The historical strength and utility of the *Drosophila* model lies in its ability to elucidate the genetic basis of complex traits, as seen in developmental biology [59] and circadian behavior [60]. This capability now extends to sleep research, offering exciting opportunities to unravel the genetic underpinnings of sleep. This research not only enhances our understanding of sleep in *Drosophila*, but also has the potential to provide valuable insights into human sleep and sleep disorders. As the scientific community works towards defining these sleep states, there is promise in leveraging *Drosophila* to map sleep state parameters to the genome, paving the way for identifying genetic factors influencing sleep states and their conservation across species.

The challenge remains to integrate these new insights with existing knowledge and to develop standardized criteria for sleep states. As methodologies are refined, the *Drosophila* model remains a powerful tool for exploring the genetic intricacies of sleep and its broader biological significance.

## Key References

- Nitz, D.A., et al., Electrophysiological correlates of rest and activity in *Drosophila* melanogaster. Curr Biol, 2002. 12(22): p. 1934-40.
  - ○This work was the first to demonstrate changes in brain electrophysiology in flies.
- Raccuglia, D., et al., Network-specific synchronization of electrical slow-wave oscillations regulates sleep drive in *Drosophila*. Current Biology, 2019. 29(21): p. 3611-3621.e3.
  - ○This study used genetically encoded voltage indicators to identify slow-wave oscillations in the fly central brain.
- Van Alphen, B., et al., A deep sleep stage in *Drosophila* with a functional role in waste clearance. Science Advances, 2021. 7(4): p. eabc2999.
  - ○The authors show that proboscis extensions correlate with a deeper sleep state, and provide evidence that the extensions remove metabolic waste.
- Tainton-Heap, L.A.L., et al., A paradoxical kind of sleep in *Drosophila melanogaster*. Current Biology, 2021. 31(3): p. 578–590.e6.
  - ○This study uncovered a wake-like or paradoxical sleep state in flies.
- Jagannathan, S.R., et al., Multivariate classification of multichannel long-term electrophysiology data identifies different sleep stages in fruit flies. Science Advances, 2024. 10(8).
  - ○This study is the first to define sleep states in flies using a combination of electrophysiological and microbehavioral criteria.

## Data Availability

No datasets were generated or analysed during the current study.
